# Distinct microbiomes underlie divergent responses of methane emissions from diverse wetland soils to oxygen shifts

**DOI:** 10.1093/ismeco/ycaf063

**Published:** 2025-04-14

**Authors:** Linta Reji, Jianshu Duan, Satish C B Myneni, Xinning Zhang

**Affiliations:** Department of Geosciences, Princeton University, Princeton, NJ 08544, United States; High Meadows Environmental Institute, Princeton University, Princeton, NJ 08544, United States; Department of the Geophysical Sciences, The University of Chicago, Chicago, IL 60637, United States; Department of Geosciences, Princeton University, Princeton, NJ 08544, United States; Department of Geosciences, Princeton University, Princeton, NJ 08544, United States; Department of Geosciences, Princeton University, Princeton, NJ 08544, United States; High Meadows Environmental Institute, Princeton University, Princeton, NJ 08544, United States

**Keywords:** wetland methane, peatland, saltmarsh, carbon-climate feedback, wetland redox dynamics, wetland microbiome, wetland management

## Abstract

Hydrological shifts in wetlands, a globally important methane (CH_4_) source, are critical constraints on CH_4_ emissions and carbon-climate feedbacks. A limited understanding of how hydrologically driven oxygen (O_2_) variability affects microbial CH_4_ cycling in diverse wetlands makes wetland CH_4_ emissions uncertain. Transient O_2_ exposure significantly stimulated anoxic CH_4_ production in incubations of *Sphagnum* peat from a temperate bog by enriching for polyphenol oxidizers and polysaccharide degraders, enhancing substrate flow toward methanogenesis under subsequent anoxic conditions. To assess whether shifts in soil microbiome structure and function operate similarly across wetland types, here we examined the sensitivity of different wetland soils to transient oxygenation. In slurry incubations of *Sphagnum* peat from a minerotrophic fen, and sediments from a freshwater marsh and saltmarsh, we examined temporal shifts in microbiomes coupled with geochemical characterization of slurries and incubation headspaces. Oxygenation did not affect microbiome structure and anoxic CH_4_ production in mineral-rich fen-origin peat and freshwater marsh soils. Key taxa linked to O_2_-stimulated CH_4_ production in the bog-origin peat were notably rare in the fen-origin peat, supporting microbiome structure as a primary determinant of wetland response to O_2_ shifts. In contrast to freshwater wetland experiments, saltmarsh geochemistry—particularly pH—and microbiome structure were persistently and significantly altered postoxygenation, albeit with no significant impact on greenhouse gas emissions. These divergent responses suggest wetlands may be differentially resistant to O_2_ fluctuations. With climate change driving greater O_2_ variability in wetlands, our results inform mechanisms of wetland resistance and highlight microbiome structure as a potential resiliency biomarker.

## Introduction

Wetlands store ~30% of the terrestrial soil carbon pool [1], despite covering only 5%–8% of the land surface [2]. Microbial decomposition of wetland carbon leads to major emissions of potent greenhouse gases, particularly methane (CH_4_) [3–5]. Wetlands are among the largest and most uncertain sources of CH_4_ to the atmosphere [6], with increasing contributions in recent years [6–8].

As wetland biogeochemistry is tightly linked to water table depth [9–11], changes in the frequency and duration of dry-wet cycles and associated oxygen (O_2_) and redox shifts can significantly alter carbon cycling and CH_4_ dynamics [11–13]. Anoxic, reducing conditions that prevail during high water table periods can be conducive for methanogenesis [14]. In contrast, drops in the water table from decreased precipitation or managed drainage can oxygenate soils, stimulating aerobic methanotrophy while simultaneously suppressing methanogenesis [15, 16]. The recent discovery of CH_4_ emissions from bulk oxic wetland soils adds further complexity to the scale of factors regulating wetland CH_4_ dynamics [17–20]. This methane paradox has been generally attributed to anoxic microsites harboring methanogens in otherwise bulk oxic soils [19, 20]. O_2_ variability, thus, imparts a complex yet strong control on wetland CH_4_ fluxes, and resolving this mechanistically is crucial for predicting global CH_4_ emissions across spatio-temporal scales.

O_2_ controls complex carbon degradation in wetland soils not only as a terminal electron acceptor (TEA) for aerobic respiration but also by regulating microbial exoenzyme activity. Microbes produce diverse classes of exoenzymes to facilitate the breakdown of complex carbon in wetlands, primarily composed of polysaccharides (e.g. cellulose, hemicellulose) and polyphenolics (e.g. lignin, tannins) [21, 22]. Polysaccharide breakdown, facilitated by hydrolases, generates simpler oligomers that are readily respired or fermented. Polyphenolics can inhibit polysaccharide hydrolysis by binding to hydrolases, effectively creating an enzyme latch on carbon degradation [23–25]. This latch is released under aerobic conditions as polyphenols are oxidized by aromatic oxidases, commonly termed phenol oxidases, which use O_2_ as a co-substrate [23–25]. Anoxic conditions, therefore, typically result in polyphenol accumulation and diminished carbohydrate hydrolysis.

This enzyme latch hypothesis was proposed as a mechanism critical for wetland carbon storage [23–25]. Many studies, however, have found contrasting evidence for the existence or strength of such a latch in various wetlands [26–28], indicating underexplored complexity in the role of polyphenols in wetland carbon fate. Furthermore, recent genomic and geochemical evidence suggests active polyphenol breakdown even under anoxia [29–31]. However, the relative magnitude of these fluxes and their importance for wetland carbon budgets remain to be ascertained.

Previous experiments on *Sphagnum* peat from a peat bog in Ward Reservation, MA (herein “Ward peat”) revealed an enzyme latch likely existed in this system as oxygenation significantly enhanced anoxic CH_4_ production in incubations [21]. Geochemical and microbial data revealed the underlying mechanisms: during the oxic period, O_2_ accelerated the breakdown of complex aromatic compounds by enriching for *Novosphingobium*, which harbor phenol-oxidases. This released the latch on hydrolysis, promoting the degradation of polymeric compounds by *Acidobacteria* (genus *Terracidiphilus*). The resulting labile carbon flux fueled fermentation by *Holophaga* during the subsequent anoxic period, enhancing substrate flux toward methanogenesis by hydrogenotrophic *Methanobacteria* [21, 32]. Transient O_2_ exposure of the bog *Sphagnum* peat thus led to a cascade of biogeochemical changes mediated by diverse microbial groups, eventually leading to ~2000-fold higher CH_4_ yields compared to continuously anoxic controls [21].

Extrapolating these observations to different wetland soils is challenging due to heterogeneous biogeochemistry and microbial dynamics [21, 33–35]. In typical *Sphagnum* peat, where phenolic compounds and carbohydrate polymers dominate organic matter [36–39], transient oxygenation can mobilize the carbon stock [26, 27, 29, 30] and enhance CH_4_ emissions. However, the magnitude of this effect may vary across soil types. For instance, abiotic reactions, such as those observed during anoxic-oxic oscillations in iron-rich soils, can also break down polyphenols (e.g. [40–44]). The enzymatic latch theory, thus, may not be universally applicable across wetlands, and consequently, the effects of O₂ shifts are likely to vary substantially.

Given diverse wetland biogeochemistry, in this study we ask the following: how does carbon cycling and methane flux respond to transient oxygenation across different wetland soils? Addressing this question is vital to understand as hydrological changes are important to the history of wetland drainage and potential future climate changes [45, 46]. Here, we assessed the response of three different wetland soils—*Sphagnum* peat from a forested fen, sediments from a mineral-soil freshwater marsh, and a saltmarsh—to brief oxygenation, and compared the results to previous work, in particular to one showing O₂-stimulated CH₄ release in *Sphagnum* peat from a temperate bog [21] and another reporting no O₂ effect on CH₄ emissions in a peat fen [47]. We hypothesized that the enzymatic latch, if present in these wetland soils, would be weakened or eliminated with O_2_ exposure, thereby amplifying anoxic CH_4_ emissions. To uncover potential mechanisms underlying wetland soil responses to transient oxygenation, we utilized a controlled laboratory incubation methodology with temporally resolved geochemical measurements. These measurements were complemented by microbial community analysis using multi-omic approaches, including 16S rRNA gene amplicon analysis, genome-resolved metagenomics, and metatranscriptomics.

## Materials and methods

### Wetland sample collection, slurry preparation, and incubation setup

Samples were collected from three biogeochemically distinct wetlands: a *Sphagnum* moss-dominated minerotrophic fen in the New Jersey Pine Barrens; a freshwater marsh in the Charles Rogers Wildlife Refuge in Princeton, NJ; and a saltmarsh in the Swan Point State Natural Area in southern New Jersey coast. The fen was located adjacent to the Bisphams Mill Creek (39.922°N, 74.591°W), in an area interspersed with Pitch Pines and various shrubs. Vegetation in the freshwater marsh (40.326°N, 74.659°W) was predominantly composed of cattails. Saltmarsh sediments were collected from the inundated margins of a shallow pond (40.0324°N, 74.0777°W), neighboring patches of *Spartina* grass.

At each location, acid-cleaned UV-irradiated polyethylene bags were filled with peat/sediments collected from the top ~10 cm beneath the litter/moss layer (if present) and transported on ice back to the laboratory. Site water (~2 l each) was also collected from each location in acid-washed Nalgene bottles. In the laboratory, slurries were prepared by blending peat/sediments (after removing plant roots and litter) with 0.2 mm-filtered site water in a 1:3 volume ratio [21]. Slurries were then flushed with ultrahigh purity 100% N_2_ (Airgas) for 15 minutes, and while continuing to flush with N_2_, 60 ml of each slurry was pipetted into acid-washed and autoclaved 160-ml serum bottles. The bottles were immediately closed with butyl rubber stoppers and crimp sealed.

For each wetland type, 24 slurry incubations were prepared. Half of these were set up as continuously anoxic controls by flushing with N_2_ gas. The remaining half were flushed with Breathing Quality air (21.5 ± 2% O_2_ and 78.5 ± 2% N_2_; Airgas). All bottles were incubated at 20 C, shaking on their side at 150 rpm in the dark. During the oxic period, half of the incubations were kept aerobic by flushing with Breathing Quality air after every 2 days. During each flush, the control incubations were flushed with 100% N_2_ gas. Oxic conditions were maintained in the O_2_-shifted incubations for a week, after which all incubations were flushed with 100% N_2_, thus establishing anoxic conditions in all. The anoxic incubations lasted 358 days, during which we tracked differences in biogeochemistry between the treatments. This involved periodic monitoring of headspace gas concentrations and destructive sampling of triplicate incubations at four timepoints (i.e. days 7, 21, 229 and 365). In a follow-up short-term incubation experiment with the Pine Barrens peat, the O_2_-shifted samples were kept aerobic for 1 month and were made anoxic afterward, with the anoxia lasting 65 days.

### Slurry sampling and geochemical measurements

Headspace gas samples were collected using a sterile Luer lock syringe (BD) to withdraw 15-ml gas into pre-evacuated, crimp-sealed amber serum vials. Gas volumes removed from the incubation headspaces were balanced by adding either 100% N_2_ or air containing 20% O_2_, as appropriate. Final gas concentrations were adjusted for headspace dilution. Concentrations of CH_4_, CO_2_, and H_2_ in the headspace samples were analyzed using a Shimadzu GC-8A gas chromatograph equipped with a Restek ShinCarbon ST column and thermal conductivity detector. Standard curves were prepared using a calibration mixture containing 1% by volume of each target species (Airgas).

On Days 7 (end of the oxic period), 21, 229, and 365, three microcosms per treatment were destructively sampled. Slurry samples for DNA/RNA (1 ml each) were aliquoted into duplicate sterile 2-ml cryotubes and stored at −80°C until extraction. A total of 100 ml each of the slurry was used for determining ferrous ion concentrations. Another 1 ml each was used for measuring dry weight and organic matter content. Remaining slurry was centrifuged for 20 minutes at 10 000 rpm to separate out the solid and liquid fractions. The supernatant was 0.2 μm-filtered and aliquoted into clean serum vials (acid washed and combusted) for dissolved organic carbon, volatile fatty acids, and phenolic content analyses. Remaining samples were saved at −20°C and later sent out to Star Labs at Ohio State University to determine carbon:nitrogen ratios (solid fraction) and dissolved anion concentrations.

Slurry pH was determined by using a waterproof pH meter (Oakton) and immersing the electrode in 1 ml of the slurry sample aliquoted into a sterile conical tube (Corning). Dry weight was determined by oven-drying the slurry samples in ceramic crucibles at 105°C to a constant weight. Percent organic matter was estimated using the loss-on-ignition method, by combusting dried slurry samples at 505 C for 3 hours. Ferrous ion and total iron concentrations were measured using the Ferrozine colorimetric assay. Standard curves were prepared by dissolving ferrous sulfate in 0.5-N hydrochloric acid. Water-extractable phenolics in the slurry supernatant was measured using the Folin–Ciocalteu colorimetric assay, using a standard curve prepared using gallic acid. Concentrations were calculated as mg/ml gallic acid equivalent. Acetate concentrations were measured on an Agilent HPLC equipped with Bio-Rad Aminex HPX-87H (300 × 7. 8 mm) and a DAD detector.

Infrared spectra of filtered supernatants were collected on a Bruker VERTEX 80v spectrometer equipped with a liquid nitrogen-cooled mercury cadmium telluride detector. See SI methods for details. IR spectra were imported to R (v4.2) and analyzed using the package ChemoSpec (v6.1.10; [48]).

All statistical analyses and plotting of the geochemical data were performed in R (v4.2; [49]), using various packages, including stats (v4.4.1; [49]) and Tidyverse (v2.0.0; [50]) core packages and add-ons.

### Nucleic acid extractions, 16S rRNA gene sequencing, and microbiome analyses

Total RNA and DNA were co-extracted from the slurry samples (Supplementary Table S1) by combining the RNeasy PowerSoil Total RNA kit with the RNeasy PowerSoil DNA Elution kit (Qiagen)*,* following manufacturer’s protocols. Nucleic acid concentrations were measured using Qubit 4 Fluorometer (Thermo Fisher), and their quality was assessed using a NanoDrop spectrophotometer (Thermo Fisher). Samples were stored at −80°C for later analyses.

The V4 region of the 16S rRNA gene was sequenced using Illumina MiSeq at the Princeton Genomics Core facility. Demultiplexed raw reads were primer trimmed using Cutadapt (v1.18; [51], and quality filtered, denoised, and merged with DADA2 (v1.26; [52]) to identify amplicon sequence variants (ASVs). ASVs were assigned taxonomy using the Silva database (r138.1; [53]). DADA2 outputs were imported into PhyloSeq (v1.38; [54]) for further analyses. See SI Methods for details on data preprocessing. The final preprocessed dataset included 28 Pine Barrens peat samples, 22 freshwater marsh samples, and 20 saltmarsh samples, including several technical replicates. All analyses were performed separately for each wetland type. See SI Methods for details on alpha diversity analysis and differential abundance testing.

### Metagenome and metatranscriptome sequencing and analyses

Nucleic acid samples passing the required quantity and quality thresholds (minimum yield of 0.5 mg and acceptable purity ratios) were used for metagenomic and metatranscriptomic sequencing (Supplementary Table S1). For each wetland type, one of the three replicates per treatment across timepoints were selected for metagenome sequencing. This amounted to nine metagenomes each per wetland type (Supplementary Table S1). Since the saltmarsh samples from later timepoints did not yield good quality DNA, only five metagenomes were obtained for these, corresponding to time zero, and two timepoints each for the two treatments (Supplementary Table S1). Metatranscriptomes were obtained for several of the PB peat and saltmarsh incubations, spanning two timepoints per treatment (Supplementary Table S1).

Paired-end sequences were obtained using the Illumina NovaSeq platform (S1 300nt flowcell) at the Princeton Genomic Core facility. QC-filtered read sets for each wetland type were co-assembled and binned into metagenome-assembled genomes (MAGs). See SI Methods for details on read QC, co-assembly, binning, mapping, and functional annotations. Relative abundance/transcription of MAGs and select functions were calculated based on length-normalized counts of mapped reads. Calculations and visualizations were performed in R (v4.2; [49]).

## Results and Discussion

### Fen-origin PB peat geochemistry resilient to the O_2_ shift

#### Headspace gases

**Figure 1:**
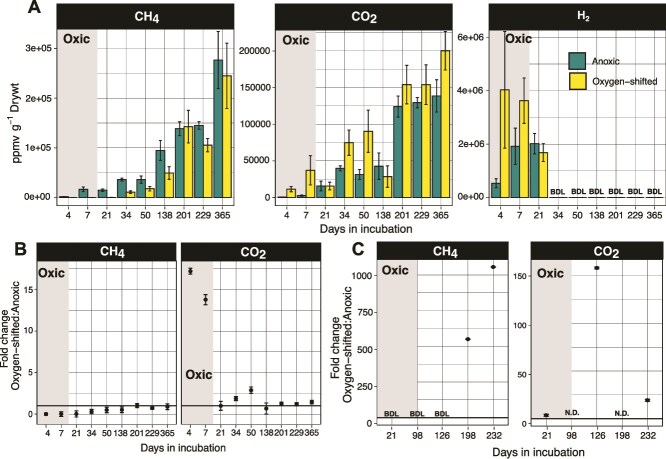
Trace gases measured in the fen-origin PB peat incubation headspaces (A and B) and compared to those in bog-origin Ward peat (C). (A) For PB peat, concentrations of CH_4_, CO_2_, and H_2_ across incubation time points. The gray rectangle indicates the oxic period for the O_2_-shifted samples. Error bars are standard errors around the mean of three replicates. (B) For PB peat, fold change in headspace CH_4_ and CO_2_ concentrations between the O_2_-shifted and continuously anoxic incubations. A fold change of 1 indicates no difference between the two treatments. (C) For Ward peat, fold change in CH_4_ and CO_2_ concentrations between O_2_-shifted and continuously anoxic incubations. Data replotted from [24]. Oxic period is indicated by the gray rectangle in each plot. N.D., not determined. BDL, below detection limit.

Transient O_2_ exposure did not stimulate CH_4_ emissions in fen-origin *Sphagnum* peat from the New Jersey Pine Barrens (herein “PB peat”), in contrast to the bog-derived “Ward peat” [21], highlighting heterogeneous responses of peat types to O_2_ shifts (Fig. 1A and B vs. C). Regardless of the oxic period duration (1 week vs. 4 weeks), there was no significant difference in CH_4_ production between O_2_-shifted samples and anoxic controls (Fig. 1A; Supplementary Fig.S1). Like in Ward peat, oxygenation delayed the onset of methanogenesis: in the O_2_-shifted incubations, CH_4_ was detected only after ~4 weeks of anoxia, compared to just 1 week in the control samples (Fig. 1A). While O_2_-shifted Ward peat incubations generated up to >1000-fold higher CH_4_ than continuously anoxic controls (Fig. 1B), overall CH_4_ yields were largely similar between the O_2_-shifted and continuously anoxic PB peat incubations (Fig. 1A), underscoring the absence of any O_2_-stimulated enhancement of methanogenesis in the latter.

O_2_ exposure also led to higher anoxic CO_2_ production in Ward peat, but not in PB peat (Fig. 1B and C). In the latter, CO_2_ production sharply increased upon oxygenation initially (Fig. 1A and B), indicating aerobic respiratory breakdown of peat carbon. The O_2_-shifted PB peat incubations yielded ~15-times more CO_2_ during the oxic period than the anoxic controls (Fig. 1B). Following the onset of anoxia, however, the two treatments were largely similar in CO_2_ production, indicating that any O_2_-induced change in CO_2_-forming metabolism was short-lived (Fig. 1A and B). This was in stark contrast to the Ward peat, which showed much higher (~20- to 150-fold) CO_2_ emissions in the O_2_-shifted incubations compared to anoxic controls long into the anoxic period following the O_2_ shift (Fig. 1C). Thus, a brief O_2_ exposure enhanced both CO_2_-forming carbon metabolism and methanogenesis in Ward peat, but not in PB peat, suggesting weaker O_2_-induced mobilization of carbon in the latter.

The headspace H_2_ concentrations provide further evidence for the lack of O_2_-stimulation of anoxic metabolism (specifically fermentation) in PB peat. Both the O_2_-shifted and control PB peat incubations showed high levels of H_2_ in the headspace during the first 3 weeks of incubation (Fig. 1A). H_2_ levels dropped below the detection limit by Week 3 and remained undetectable for the remainder of the experiment (Fig. 1A). In contrast, significant amounts of headspace H_2_ were detected in the Ward peat incubations even at the final incubation timepoint (Day 232, 134 days anoxic; Supplementary Fig. S2; [21]). Additionally, acetate, another key product of microbial fermentation, accumulated at millimolar levels in the Ward peat (up to ~2.5 mM in O_2_-shifted Ward peat and ~ 7 mM in the anoxic controls; [21]). In contrast, no measurable acetate was detected in the PB peat incubations at any timepoint during the incubation (detection limit 560 mM). These observations suggest that fermentation was either not particularly active or that its byproducts were actively recycled in PB peat. In the Ward peat, accumulation of H_2_ and acetate over time suggests that O_2_-exposure increased the substrate availability for fermenters, but methanogens likely could not keep up with the increasing substrate flux, leading to decoupling of production and consumption processes.

#### Aqueous phase geochemistry

**Figure 2:**
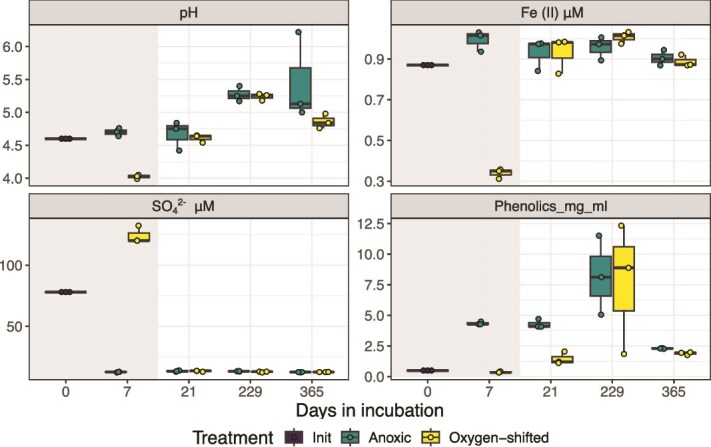
Changes in wetland soil geochemistry with the O_2_ shift. Key geochemical variables measured in PB peat across timepoints in the experiment. Oxic period is indicated by the gray rectangle. Three replicate measurements are included in each boxplot (overlayed dots depict the actual values). Phenolics concentrations were measured as mg/ml gallic acid-equivalent.

O_2_ exposure further led to a decline in pH and Fe(II) concentrations in the PB peat incubations, which coincided with a sharp increase in sulfate (SO_4_^2−^) levels (Fig. 2), likely derived from the oxidation of reduced organosulfur compounds that can be abundant in peat [55] or iron sulfides [56, 57]. Fe(II) and SO_4_^2−^ levels were reestablished to prior values during anoxia (Fig. 2), potentially indicating the coupled effect of microbial alternative respirations (sulfate/Fe(III) reduction) and the regeneration of reduced iron–sulfur compounds. At least at the very beginning of the anoxic period in the O_2_-shifted peat, SO_4_^2−^ and Fe(III) may have served as alternative electron acceptors for microbes. The measured SO_4_^2−^ levels, however, remained well-below the limiting concentration for microbial sulfate reduction (300 mM; [55, 58]; Fig. 2), and therefore, the importance of microbial sulfate respiration remains unclear.

Collectively, the geochemical observations indicated carbon degradation in PB peat to be largely unaffected by the O_2_ shift, likely due to a weak enzymatic latch in this peat. This is further evidenced by the lack of measurable phenolics in the initial PB peat sample (Fig. 2). Total phenolics were ~ 2- to 5-fold higher in the anoxic controls compared to the O_2_-shifted samples on Days 7 and 21 (Fig. 2), indicating some O_2_-mediated breakdown of polyphenols in these samples. Following the oxic period, FTIR data indicated the mobilization of small organic molecules, including aromatics (C=C at 1627, 1594, and 1493 cm^−1^), amides (amides I and II bands at 1655 and 1579 cm^−1^, respectively), and aliphatics (C-H between 2700 and 3000 cm^−1^), which were absent in the anoxic controls and diminished at later timepoints, suggesting microbial consumption (Supplementary Fig. S3). The anoxic and O_2_-shifted samples had largely similar IR spectra at later timepoints during anoxia (Supplementary Fig. S3), suggesting a reset of soluble organic matter composition following oxygenation. This contrasts starkly with the Ward peat, where FT-ICR-MS data revealed major changes in organic matter composition, notably significant O_2_-enhanced removal of aromatic compounds [21].

Overall, geochemical measurements point to differential controls on microbial carbon metabolism in the PB peat compared to those previously identified in the Ward peat. Even a relatively short period of O_2_ exposure enhanced CH_4_ release during anoxia in Ward peat (e.g. ~55-fold increase in anoxic CH_4_ emissions following a week of O_2_ exposure [21]). The apparent absence of a similar effect in PB peat (Fig. 1A), even with a longer, 30-day period of O_2_ exposure (Supplementary Fig. S1) points to fundamentally different O_2_ controls on microbial metabolisms and carbon cycling in these two *Sphagnum* peats. In addition, the lack of accumulation of fermentation byproducts in PB peat indicates either a tight coupling of fermentation with methanogenesis or alternative anaerobic respiration of organic matter in this peat.

#### Fen-origin PB peat microbiome largely resilient to the O_2_ shift

**Figure 3:**
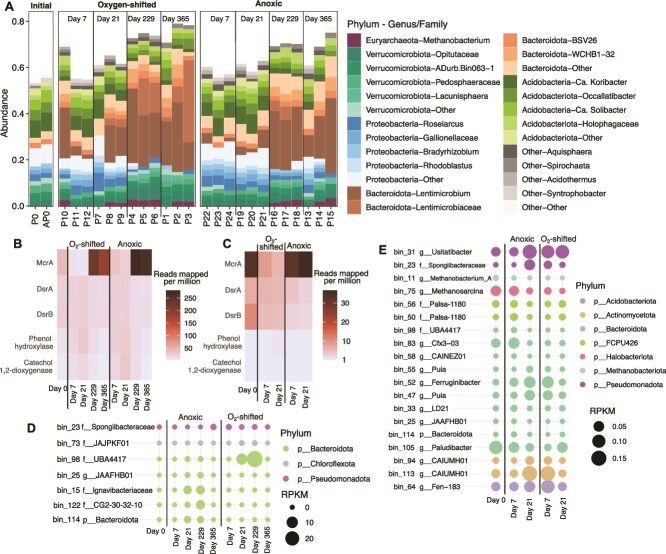
PB peat microbial community response to the O_2_ shift. Microbial community composition across incubation timepoints assessed using the V4 region of 16S rRNA gene. (A) Top 5 phyla comprising >50% of the community in the PB peat microbiome. Dominant genus or family-level lineages are also highlighted for each phylum. Gray box in the “oxygen-shifted” panel indicates replicate samples at the end of the oxic period. (B) Relative abundances of selected functions in the PB peat metagenomes. McrA, methyl coenzyme reductase; DsrA and DsrB, sulfite reductase subunits A and B. (C) Relative abundance of the selected functions in the PB peat metatranscriptomes. MAGs identified as differentially abundant (D) and active (C) between O_2_ treatments in PB peat. For each MAG, taxonomic identity at the genus, family, or phylum-level has been presented. RPKM, reads mapped per kilobase of genomes per million total reads.

Microbial community structure and its response to the O_2_ shift in the fen-origin PB peat contrasted strongly with that of Ward peat. O_2_-stimulation led to significant changes in the Ward peat microbiome [21, 32]. The PB peat microbiome, in contrast, was largely resilient to the O_2_ shift, as the two treatments were similar in community composition throughout the experiment (Fig. 3A). As the experiment progressed, the PB microbiome shifted from a community dominated by *Proteobacteria* and *Acidobacteriota* to one dominated by *Bacteroidota* (Fig. 3A). These changes were, however, independent of the O_2_ shift, as they were consistent between anoxic and O_2_-shifted samples.

### Notable taxa and functions associated with a weaker “latch” in PB peat

Key taxa observed to be vital players in the O_2_-stimulated CH_4_ emissions in Ward peat were notably rare in PB peat. In Ward peat, three major microbial groups were identified as key players in the O_2_ response: *Novosphingobium* harboring aromatic oxidases, *Acidobacteria* (specifically, the genera *Holophaga* and *Terracidiphilus*) capable of polysaccharide hydrolysis and fermentation, and *Methanobacterium*, a hydrogenotrophic methanogen [21, 32]. These taxa were relatively rare (overall abundance <5% of the community) in the PB peat microbiome (Fig. 3A; Supplementary Fig.S4). The relative abundance of *Novosphingobium* indeed increased immediately following the O_2_ exposure, however, they still only comprised <2% of the total community (Supplementary Fig. S4). Similarly, the primary methanogen in the community, *Methanobacterium*, also did not increase in relative abundance during anoxia in O_2_-shifted PB peat unlike in Ward peat (Fig. 3A).

While O_2_ exposure did not significantly alter the PB peat microbiome, subtle and short-lived compositional changes were observed. Alpha diversity increased sharply with O_2_ exposure (Supplementary Fig. S5), as diverse microbes likely exploited the new niche for aerobic respiration. This effect was relatively short-lived as the O_2_-shifted samples progressively became less diverse over time, significantly lower than anoxic controls at later timepoints (Supplementary Fig. S5; Wilcoxon Rank Sum test with FDR adjusted *P*-values <2e-16 for all). In comparison, the continuously anoxic controls showed largely invariable ASV richness throughout the experiment (Supplementary Fig. S5). ANCOM differential abundance testing uncovered 24 ASVs in the PB peat microbiome as differentially abundant between the O_2_-shifted and continuously anoxic treatments (q-value <0.001; Supplementary Fig. S6). However, most of these ASVs had log fold change values <1 (with the maximum being 1.5; Supplementary Fig. S6), suggesting that the abundance differentials were rather small. Both treatments showed a reduction in community evenness over time (as measured using the Inverse Simpson index), suggesting an increasingly uneven distribution of microbial populations among the ASVs (Supplementary Fig. S5). In other words, while the overall community richness decreased over time, certain ASVs (i.e. “species”-level lineages) became increasingly dominant.

Microbial functional profiles also highlighted the differential response of the two peats to O_2_ shift: aromatic oxygenases (phenol hydroxylases and catechol 1,2-dioxygenases) enriched during the oxic period in Ward peat [21] were of notably low abundance in PB peat and did not respond to the O_2_ shift (Fig. 3B and C). This agrees with the FTIR data showing minimal changes in aromatic compound transformations and only a modest accumulation of likely oxidized aromatics in the aqueous phase following oxygenation (Supplementary Fig. S3). Sulfite reductase (DsrAB) transcripts were relatively more enriched in the O_2_-shifted PB peat than in the anoxic controls (Fig. 3B and C), which coincided with the higher levels of sulfate measured in the O_2_-shifted samples at the end of the oxic period (Day 7; Fig. 2). Methanogenesis, as expected, was more enriched in the continuously anoxic controls compared to the O_2_-shifted samples (using the marker gene methyl coenzyme M reductase, *mcrA*; Fig. 3B and C).

Assembling metagenome contigs into draft genomes (i.e. MAGs) allowed a more nuanced look at the differentially abundant and active lineages across treatments PB peat. We obtained 72 medium- to high-quality MAGs (i.e. >70% complete; <10% contamination) from the metagenomes (Supplementary Table S2). Differential abundance testing [59] revealed 7 MAGs as being differentially abundant (Fig. 3D), and 19 as differentially transcribed across treatments (Fig. 3E). Lineages within major phyla appeared to respond differentially to the O_2_ shift, suggesting diverging niches among related organisms. For example, while five of the six differentially abundant *Bacteroidota* MAGs were more enriched in the anoxic controls, one was more abundant in O_2_-shifted samples (Fig. 3D). Functional comparisons did not reveal any discernable metabolic features between these MAGs. The *Pseudomonadota* MAG bin_23 (a novel genus within family *Spongiibacteraceae*) enriched during the oxic period (Day 7; Fig. 3D and E), harbored pathways for O_2_-dependent degradation of aromatic compounds (Dataset 1). While most of the differentially transcribed MAGs are facultatively anaerobic heterotrophs (Dataset 1), several stood out for their alternative respiration capabilities. Specifically, bin_31 (*Pseudomonadota* genus *Usitatibacter*) and bin_64 (*Acidobacteriota* genus Fen-183), differentially abundant in the O_2_-shifted metatranscriptomes (Fig. 3E), have the potential for sulfate reduction (Dataset 1). Bin_31 additionally has the potential for iron oxidation (Supplementary Table S3). Their increased transcription during the oxic period thus coincides with the initial decrease in Fe(II) and surge in sulfate (Fig. 2).

### Freshwater marsh and saltmarsh responses to O_2_ shift

**Figure 4:**
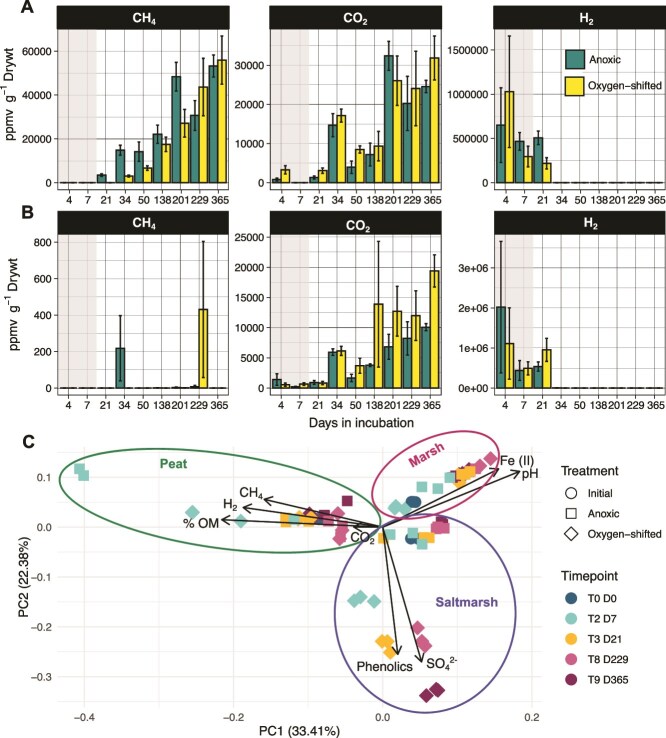
Geochemical responses of FW-marsh and saltmarsh to O_2_ shift. CH_4_, CO_2_, and H_2_ measured in the incubation headspaces across timepoints in FW marsh (A) and saltmarsh (B). The gray rectangle indicates the oxic period for the O_2_-shifted samples. Error bars are standard errors around the mean of three replicates. (C) PCA of key geochemical variables measured over the course of the experiment. Shapes indicate different treatments. Ellipses are drawn to indicate each wetland type.

To compare the sensitivity of different types of wetlands to O_2_ shifts, we repeated the incubation experiments with sediments collected from a freshwater marsh (herein “FW marsh”) and saltmarsh. CH_4_ was detected in the FW marsh incubation headspaces only after 3 weeks of anoxic incubation (compared to 1 week of anoxic incubation of PB peat). CH_4_ yield was generally an order of magnitude lower than in PB peat (Fig. 1A and4A). CH_4_ production in the O_2_-shifted samples was initially lower compared to the controls; however, over time, similar quantities of CH_4_ were measured in both treatment headspaces (Fig. 4). Thus, similar to PB peat, transient O_2_ exposure had no significant effect on CH_4_ release during the subsequent anoxic period in FW marsh (Fig. 4A). Initially, CO_2_ production in the O_2_-shifted FW marsh incubations were slightly higher than that in anoxic controls, but the difference was increasingly insignificant over time (Fig. 4A).

In contrast to both PB peat and FW marsh, the O_2_-shifted saltmarsh incubations released higher amounts of CO_2_ (1.6- to 3.8-fold) during the anoxic period than continuously anoxic controls (Fig. 4B). Notably, this difference was only observed with long (>6 weeks) anoxic incubation of the O_2_-shifted saltmarsh samples; during and immediately following the oxic period, both treatments released similar amounts of CO_2_ (Fig. 4B). Relatively small amounts of CH_4_ were detected in a few of the saltmarsh incubations at select timepoints (Fig. 4B); however, the levels dropped to below detection limit at later timepoints, suggesting active turnover of this CH_4_ (Fig. 4B).

Geochemical properties of the slurries changed quite significantly upon oxygenation in all three wetland types (Fig. 4C; Supplementary Fig.S7). These changes, however, were rather short-lived in both PB peat and FW marsh as both treatments converged over time (Figs 2 and 4C; Supplementary Fig.S7). Saltmarsh geochemistry, however, was more persistently altered upon O_2_ exposure: samples in the two treatments continued to diverge in their measured geochemical properties even after several months of anoxic incubations (Fig. 4C; Supplementary Fig.S7). These changes were likely driven by the significant drop in pH (about 6.5 in the initial sample to 4 in the O_2_-shifted sample; Supplementary Fig. S7), and ~2-fold increase in sulfate levels upon oxygenation (Supplementary Fig. S7).

### Microbiome responses to O_2_ shifts in the freshwater marsh and saltmarsh incubations

**Figure 5:**
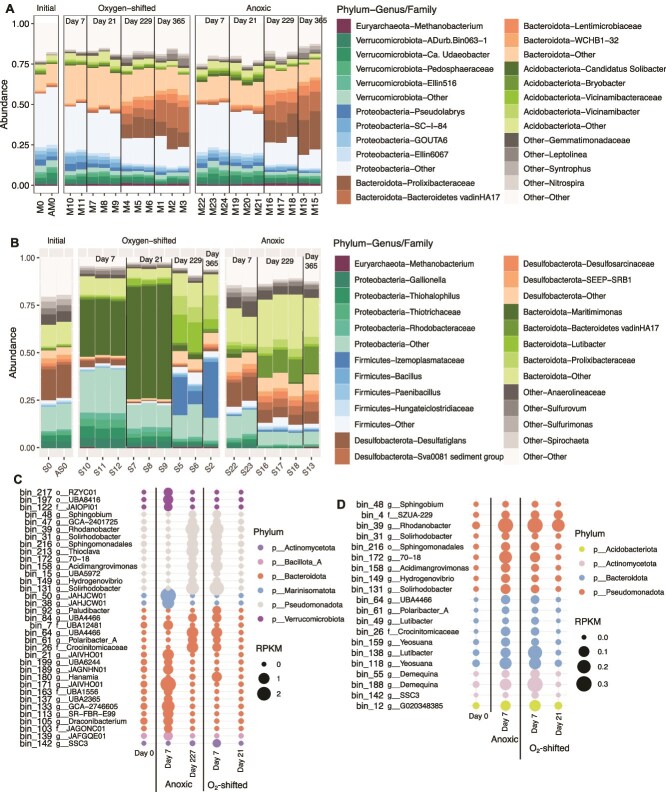
Microbiome response to O_2_ shift in FW-marsh and saltmarsh. Microbial community composition across time points in FW-marsh (A) and saltmarsh (B), assessed using the V4 region of 16S rRNA gene. Top 5 phyla comprising >50% of the communities are highlighted in each plot. Dominant genus or family-level lineages are also indicated for each phylum. (B) MAGs identified as differentially abundant (D) and active (C) between O_2_ treatments in the saltmarsh incubations. For each MAG, taxonomic identity at the genus, family, or phylum-level has been presented. RPKM: Reads mapped per kilobase of genomes per million total reads.

FW marsh microbiome response to O_2_ shift was strikingly similar to that in PB peat, with both communities transitioning form *Proteobacteria*-dominated to *Bacteroidota*-dominated over time (Fig. 5A). As in the case of PB peat, these changes did not reflect any effect of the O_2_-pretreatment, as O_2_-shifted and anoxic samples were compositionally similar across timepoints (Fig. 5A). These observations further align with the minimal changes in geochemistry upon oxygenation as discussed above (Fig. 4A and C).

The saltmarsh microbiome, in contrast, changed significantly upon O_2_-addition (Fig. 5B), as expected based on the diverging geochemical properties of the control and O_2_-shifted samples (Fig. 4C). The controls were dominated by *Desulfobacterota* and *Bacteroidota* throughout the incubation period, with little compositional changes with time (Fig. 5B). In contrast, the O_2_-shifted samples were differentially enriched in *Proteobacteria* and specific lineages of *Bacteroidota*, with *Firmicutes* becoming abundant toward the final timepoints (Fig. 5B). In particular, the genus *Maritimimonas* (phylum *Bacteroidota*) significantly increased in relative abundance upon oxygenation and remained dominant into early phase of the anoxic period (Fig. 5B). With prolonged anoxia, a different *Bacteroidota* genus *Lutibacter* became increasingly more abundant in the O_2_-shifted samples, along with the *Firmicutes* family Izemoplasmataeceae (Fig. 5B). In contrast to PB peat, much higher log fold change values (up to 4.5 and − 3.5) were observed for the differentially abundant ASVs in the saltmarsh incubations (Supplementary Fig. S6c).

Several phylogenetically novel MAGs were obtained from the saltmarsh metagenomes, including one representing a new phylum-level lineage (Supplementary Table S2), with largely heterotrophic metabolism (Dataset 2). Aligning with the community composition patterns observed for the ASV data, many of the MAGs were conspicuously differentially abundant across the two treatments (Fig. 5C and D). The altered geochemistry persisting long after O_2_ exposition in the saltmarsh (Fig. 4C) likely promoted differential enrichment of microbial taxa in these incubations.

### Broader implications

#### Microbial and geochemical factors regulating wetland response to O_2_ shifts

**Figure 6:**
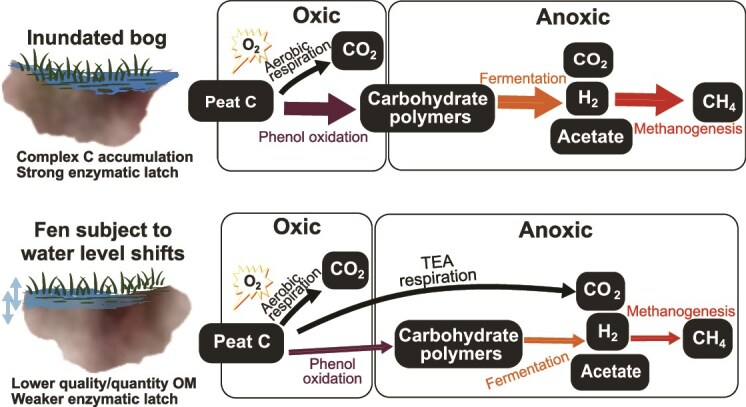
Schematic diagram of microbially-mediated carbon flow with O_2_ shift in the (A) Ward sphagnum peat compared to (B) PB sphagnum peat. In contrast to Ward peat, transient oxygenation does not lead to a surge in anoxic CH_4_ production in PB peat, likely due to (i) the PB fen being subject to fluctuations in water (and thus O_2_) levels, favoring a microbiome with lower sensitivity to O_2_ variability, and (ii) alternative TEAs redirecting carbon flow away from CH_4_. The width of the arrows presents the importance of relevant metabolisms.

Several studies have reported accelerated carbon degradation (i.e. CO_2_ emissions) in response to droughts or redox shifts in wetlands [9, 21, 60]. While heterogeneity in these responses across wetlands is well documented (reviewed in [61]), the mechanisms remain poorly understood. Our results highlight such variable responses, even in peat dominated by the same plant type *Sphagnum*, and provide insight into the underlying mechanisms. The lack of O_2_-stimulation of carbon mobilization in PB peat was exemplified by (i) minor compositional shifts in the microbiome (Fig. 3), (ii) lack of enrichment of polyphenol oxidizers (Fig. 3B and C), and (iii) the lack of accumulation of fermentation byproducts (H_2_ and acetate). Overall, microbial carbon degradation appears to have followed significantly different routes in this peat compared to the *Sphagnum* bog peat studied previously. These differences likely arise from various factors, including microbiome compositional differences, variability in alternative electron acceptors, and the larger ecological context of each peat system (Fig. 6). Hydrologic history of the peat system further impacts the quantity and quality of organic matter, which may be an additional factor controlling response to O_2_ shifts.

The environmental context of the two peats were substantially different: while the Ward peat originated from a peat bog, the PB peat originated from a fen adjacent to a freshwater creek. Given its proximity to the creek, the PB peat system likely experiences more frequent changes in water table levels compared to the Ward peat bog, which likely imparts a higher degree of tolerance for hydrological and O_2_ variability. In addition, such frequent aeration cycles may deplete readily oxidized phenolic compounds in peat OM, reducing O_2_-stimulation of carbon mineralization. These results are comparable to the observations in [47], where dry-wet manipulations in a Boreal minerotrophic fen did not stimulate phenol oxidase activity or enhance CH_4_ production, despite an initial surge in CO_2_ production. This study also reported an increase in sulfate, Fe(III), and nitrate concentrations upon soil drying. While nitrate levels in the PB peat was below detection limit throughout, the increase in sulfate concentrations we observed (~112 uM) were largely comparable to those reported in [47] (>100 uM). At least part of the carbon flow in PB peat may have thus been directed toward anaerobic respiration, specifically sulfate reduction, which is further supported by the relatively higher expression of Dsr genes and putative sulfate reducers in the PB peat metatranscriptomes (Fig. 3B and C). These results also compare to the recent observation that carbon decomposition in northern peatlands is likely dominated by TEA respiration rather than methanogenesis [62].

Geochemical and microbial data thus indicate that unlike in Ward peat, phenolic compounds, or lack thereof, may not comprise a strong latch on PB peat carbon degradation. The multiple lines of microbial data we present here—compositional structure, functional potential, and putative activity—reveal that the strong resistance of the PB peat microbiome to O_2_ shifts is key to C storage in this system, in stark contrast to Ward peat. In agreement with field-based studies [47], our observations collectively suggest that O_2_ fluctuations in minerotrophic fen systems that experience periodic hydrologic fluctuations are unlikely to stimulate CH_4_ emissions. The lower sensitivity of the microbiomes and carbon dynamics to O_2_ variability in such systems suggests greater carbon stock stability under projected global change scenarios.

Additionally, the strikingly dissimilar response of the saltmarsh sediments to transient oxygenation points to varying microbial community compositions and ionic interactions shaped by salinity and sulfate levels, which may cause divergent responses of brackish coastal wetlands to O_2_ shifts. Systems subjected to tidal flushing and associated periodic changes in O_2_ levels may, however, be more buffered against O_2_ variability.

#### Wetland resistance to O_2_ shifts in the context of global change

Our observations suggesting differential resistance of wetlands to O_2_ shifts has important implications for wetland management under global change. Compared to bogs, mineral soil wetlands (e.g. FW marsh), and minerotrophic peatlands supporting alternative respiration (e.g. PB peat) are likely more resistant to O_2_ shifts. Partly, this resistance could stem from microbial community adaptation to redox shifts. Such adaptation potential may be absent in permanently anoxic sediments of saltmarshes, as observed here. While the validity of these results to field settings remains to be assessed, our observations indicate potentially major, persistent changes in saltmarsh biogeochemistry due to redox variability from coastal restoration or sea-level rise. Such effects may be particularly pronounced in systems with relatively steady hydrologic conditions, such as prolonged dryness or persistent inundation.

Our observations further point out specific microbial taxa as potential indicators of wetland resiliency under hydrological variations and indicate that microbiome data can inform predictions of wetland behavior upon redox shifts. The results also underscore the need to assess wetland resiliency in the context of their divergent ecological settings. Such careful characterization of the environmental heterogeneity is essential for accurately scaling up laboratory observations to predictive global models of peatland methane emission trajectories.

## Supplementary Material

Wetland-MS-Submission_ISMEcomm_SI_v1_ycaf063

SI_tables_ycaf063

Dataset_1_peatMAGs_DRAM_ycaf063

Dataset_2_saltmarshMAGs_DRAM_ycaf063

## Data Availability

All sequence data have been deposited in the NCBI Sequence Read Archive under the BioProject accession PRJNA1123272 (data will be publicly available upon publication). All other datasets, including geochemical data and 16S rRNA gene amplicon analysis files, and R codes used for analyses and for generating the figures are available at the GitHub repository (https://github.com/Linta-Reji/Reji2024_wetland_O2shifts), with large files uploaded to Zenodo (doi: 10.5281/zenodo.14510955).
