# Correction to: Distinct microbiomes underlie divergent responses of methane emissions from diverse wetland soils to oxygen shifts

**DOI:** 10.1093/ismeco/ycaf214

**Published:** 2026-06-22

This is a correction to: Linta Reji, Jianshu Duan, Satish C B Myneni, Xinning Zhang, Distinct microbiomes underlie divergent responses of methane emissions from diverse wetland soils to oxygen shifts, *ISME Communications*, Volume 5, Issue 1, January 2025, https://doi.org/10.1093/ismeco/ycaf063

The following changes have been made to the originally published manuscript. Xinning Zhang has been added to be marked co-corresponding author.

