# Supplementary material for: Distinct microbiomes underlie divergent responses of methane emissions from diverse wetland soils to oxygen shifts
Source: ISME Commun. 2025 Apr 14;5(1):ycaf063. doi: 10.1093/ismeco/ycaf063 (PMC12075768; doi:10.1093/ismeco/ycaf063)
Supplement: Wetland-MS-Submission_ISMEcomm_SI_v1_ycaf063 [file wetland-ms-submission_ismecomm_si_v1_ycaf063.pdf]

Distinct Microbiomes Underlie Divergent Responses of Methane Emissions from Diverse Wetland soils to Oxygen Shifts

Running head: Wetland microbiome response to oxygen shifts

Linta Reji[1,2,3], Jianshu Duan[1], Satish Myneni[1], Xinning Zhang[1,2]
[1] Department of Geosciences, Princeton University, NJ
[2] High Meadows Environmental Institute, Princeton University, NJ
[3] Current address: Department of the Geophysical Sciences, The University of Chicago, IL

Supplementary Methods

Infrared spectra collection

Aliquots (1 µL) of filtered supernatants following slurry centrifugation, were added to the surface of a diamond ATR (attenuated total reflectance) crystal mounted on a Golden Gate ATR sample stage (Specac), and the solution was dried under moisture- and CO_2-free air (Spectra 30 FTIR purge gas generator, Parker Balston). This step was repeated 5 times until a thin film was formed. The sample spectrum was measured as the average of 2000 scans at a scan velocity of 80 kHz with a 5 mm aperture setting. A blank spectrum was collected immediately before each sample spectrum and subtracted. All spectra were collected at 4 cm^{-1} resolution. Baseline corrections were performed on the ATR spectra using the OPUS software (Bruker).

16S rRNA MiSeq data processing and analyses

DADA2 outputs (i.e., taxonomy and ASV count tables) were imported to Phyloseq (1) for further analysis. Eukaryotic, including mitochondrial and chloroplast, sequences were filtered out. During pre-processing, rarefaction curves were calculated separately for each wetland type to determine outliers to discard from analyses. As a result, samples without a plateauing rarefaction curve were discarded.

Alpha diversity (Chao1 and Inverse Simpson) was measured using the plot_richness function in Phyloseq. The tax_fix function in microViz (v0.10.2; (2)) was used to fix missing values in the taxonomy tables. Bar plots of relative abundances were then obtained using the microshades (v1.10; (3)) package. Differential abundance testing was performed at the genus level by using Analysis of Composition of Microbiomes with Bias Correction (4,5) as implemented in the ANCOMBC package (v1.4).

Metagenome and metatranscriptome analyses

Demultiplexed metagenomic reads were quality filtered using FastQC (v0.11.9; (6)) and Trimmomatic (v0.39; (7)). Anvi'o (v7.1; (8)) was used for assembly, mapping, and contig functional assignments. Metagenomes for each wetland type were co-assembled using MEGAHIT (--min-contig-len 1000; v1.2.9; (9,10)). Bowtie 2 (v2.3.5; (11)) was used to map

reads to the assembled contigs. Contig functional annotations were obtained by using the functions "anvi-run-hmms" and "anvi-run-ncbi-cogs". Demultiplexed metatranscriptomes were quality filtered using FastQC (v0.11.9; (6)) and cutadapt (v2.10; (12)). QC-filtered reads were mapped to co-assembled metagenomic contigs for each wetland type. Mapping was performed using bowtie2 (v2.3.5; (11)) in Anvi'o (v7.1; (8)). Metagenome contigs were binned using MetaBAT 2 (v1.12.1; (13)), MaxBin 2 (v2.2.7; (14)), and CONCOCT (v1.1.0; (15)). Bin refinement was carried out using the bin_refinement module in MetaWRAP (v1.2; (16)). Prodigal (v2.6.3; (17)) was used to obtain amino acid sequences of gene calls for each bin. These were uploaded to the GhostKoala server (18) to obtain KO annotations. Refined bins along with the corresponding KO annotations were exported into the merged Anvi'o profile as a collection. Relative distributions of assembled genomes and predicted functions across the metagenomes and metatranscriptomes were estimated using the "anvi-summarize" function. Additional functional annotations for the assembled genomes were obtained via DRAM (19) and RASTtk (20) within the KBase platform (21).

Supplementary Figures

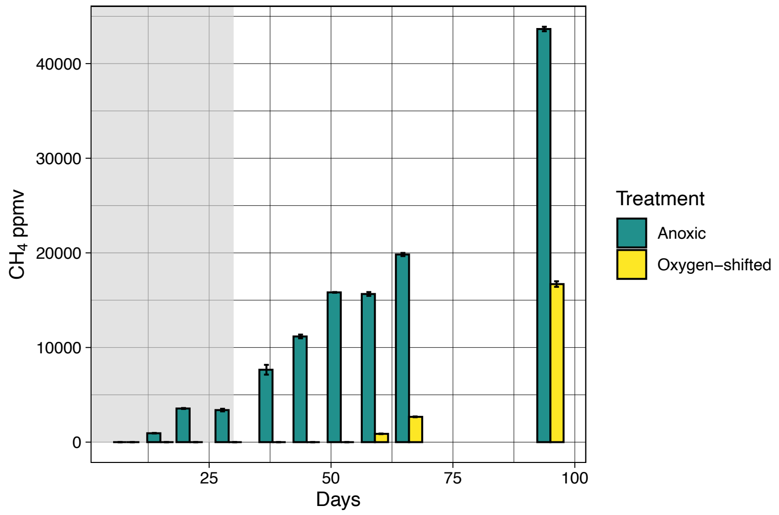

Figure S1: Headspace methane concentrations in the PB peat incubations when PB peat was exposed to a longer period of oxygenation (i.e., 4 weeks). Error bars are standard deviations around the mean of three replicates.

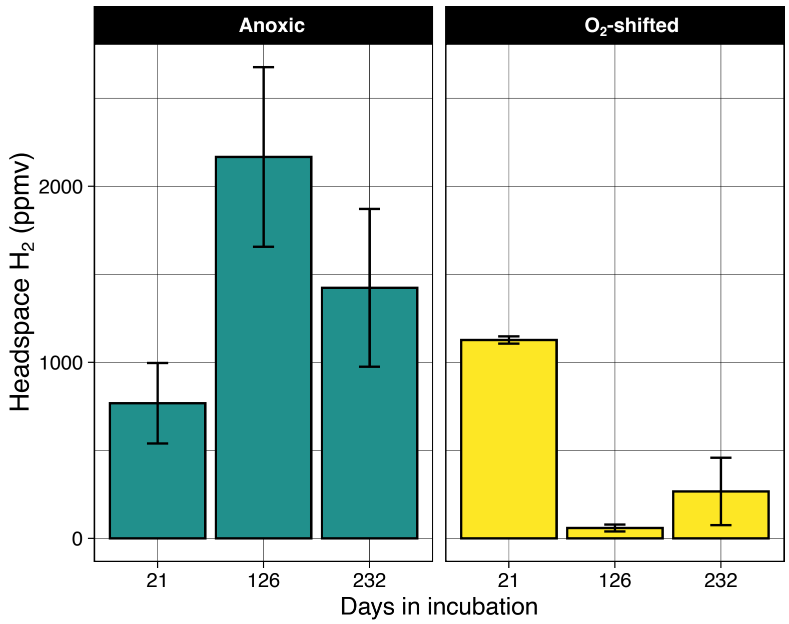

Figure S2: H_2 Measured in Ward peat incubations. Error bars are standard errors around the mean of three replicates. Data re-plotted from Wilmoth et al., 2021(22).

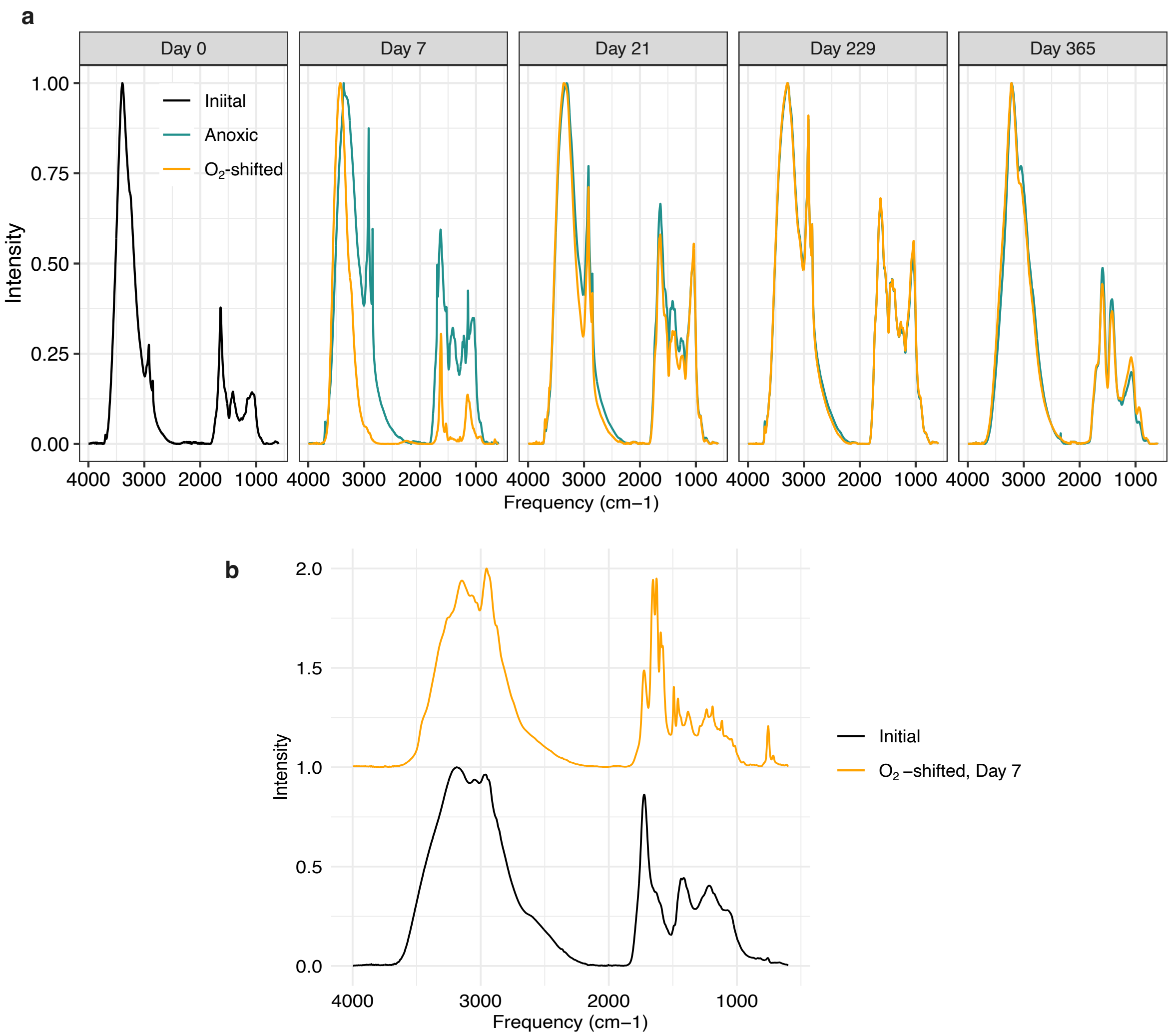

Figure S3: FTIR spectra of PB peat slurry extracts (a) and those after desalting for the Day 7 samples (b). The Day 7 samples were desalted and compared due to significant salt interferences (indicated by the strong O–H bending band at 1620 cm-1) in the O2-shifted sample. Bottom panel (b): initial sample and O_2-shifted Day 7 samples were re-ran after desalting: spectra are similar to the original run, but show greater amounts of C-H bonds (carbohydrates) in the O_2-shifted sample (~ 1100-1600 nm region), indicating greater amounts of labile carbon in solution on Day 7. Samples on Day 365 probably highly degraded for both treatments.

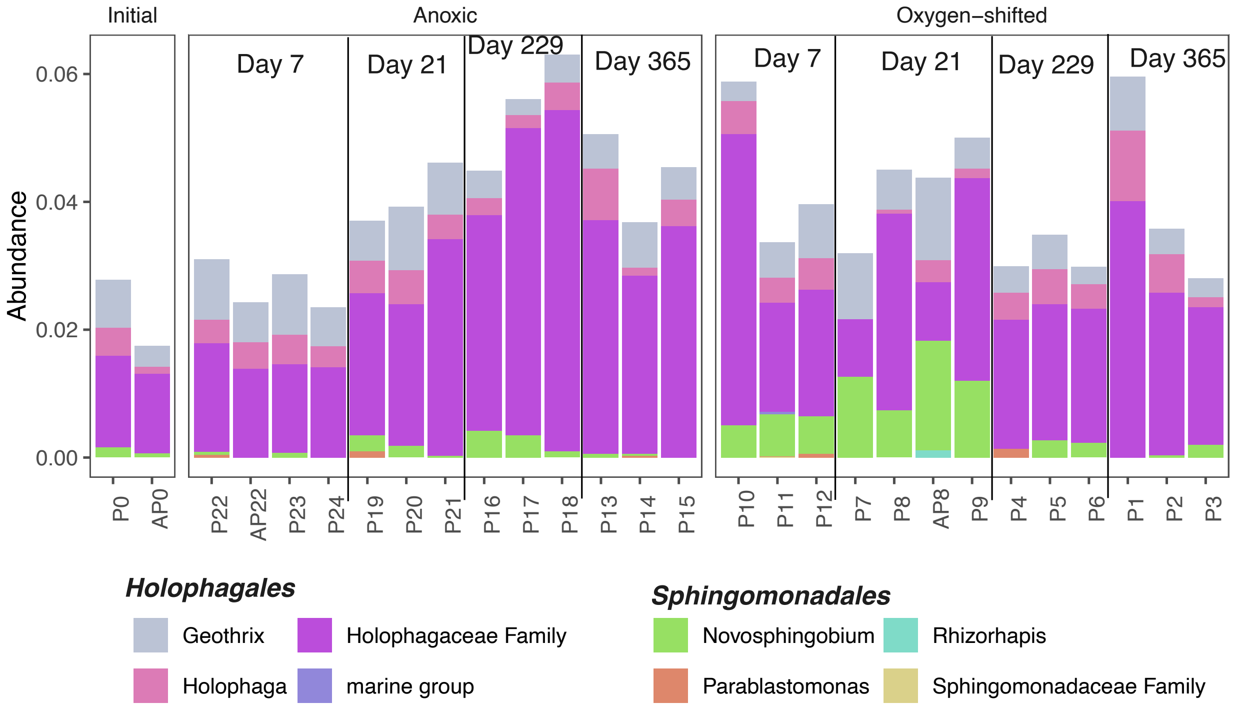

Figure S4: Relative abundance of selected lineages in the PB peat across timepoints. These lineages were identified as key indicator taxa for O_2-stimulation of methanogenesis in the Ward *Sphagnum* peat.

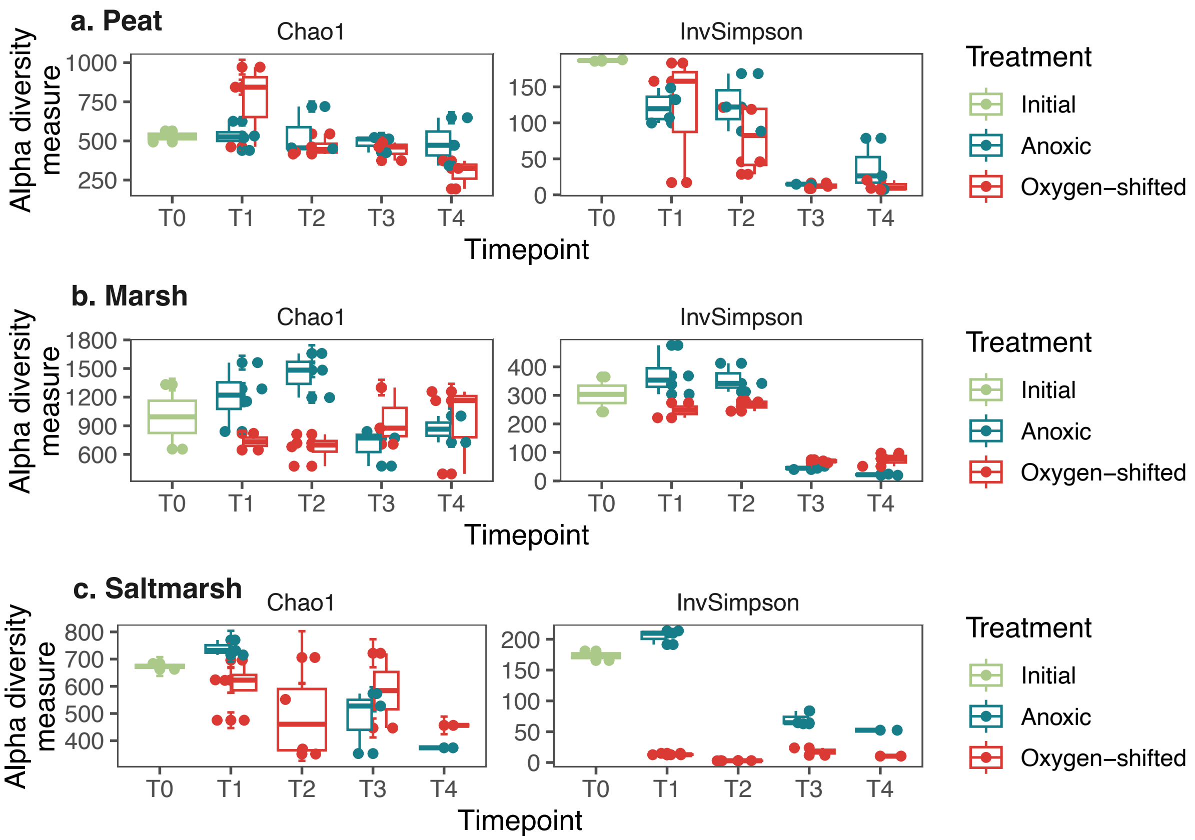

Figure S5: Estimates of microbial community alpha diversity (i.e., Observed number of ASVs and Inverse Simpson index) in the incubations across time for (a) peat, (b) marsh, and (c) saltmarsh. The filtered data used for alpha diversity estimation did not contain singletons. The data were rarefied to even depth before diversity estimation.

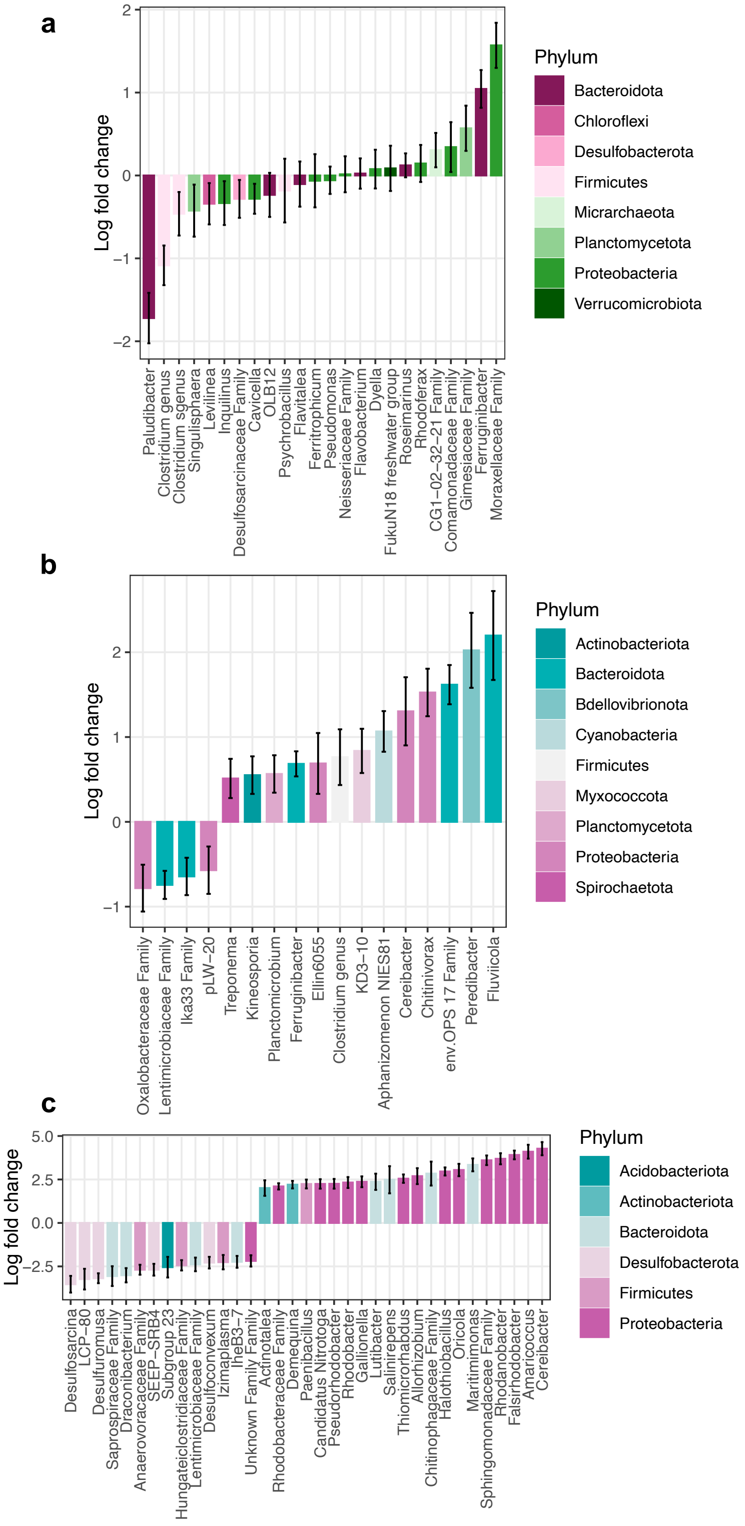

Fig. S6: Summary of the differentially abundant ASVs between treatments in (**a**) PB peat, (**b**) FW marsh, and (**c**) saltmarsh. ANCOM-BC was used for differential abundance analysis. Since the microbiome changed substantially between treatments for saltmarsh, only those with a fold change of at least 2 are included in (**c**).

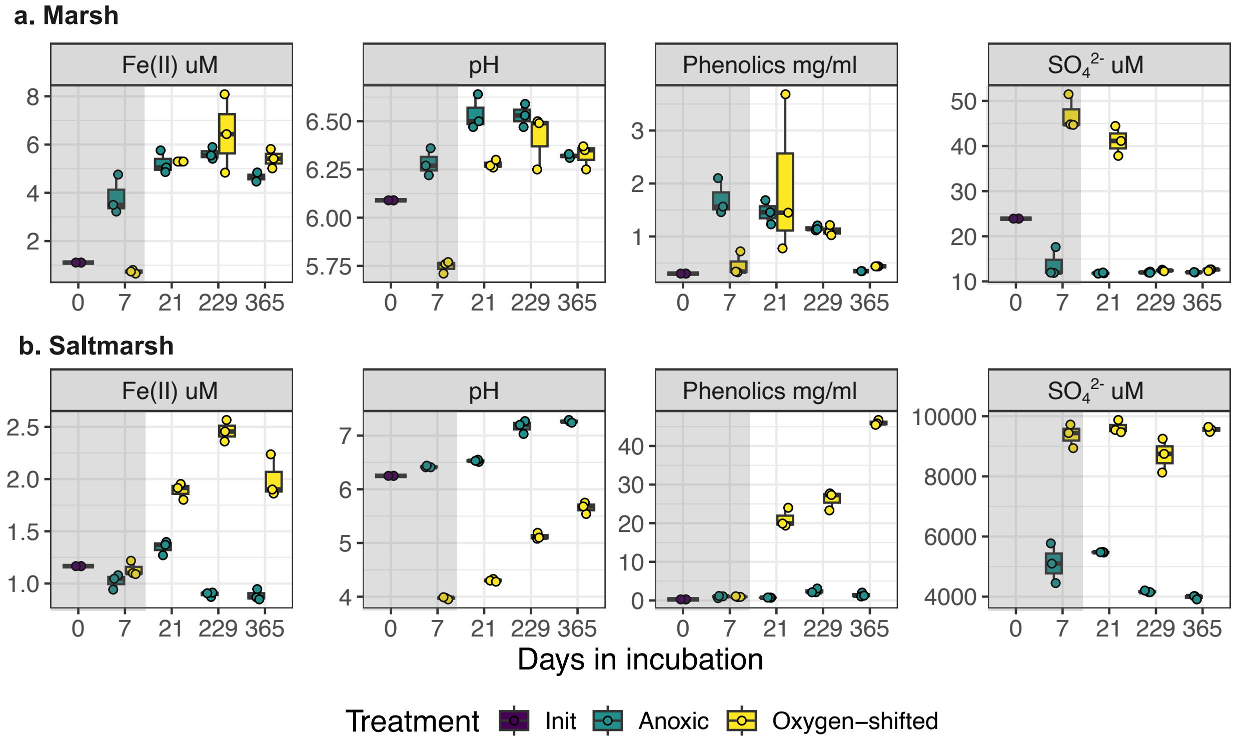

Figure S7: Key geochemical variables measured in FW marsh and saltmarsh incubations across timepoints in the experiment. Oxic period is indicated by the grey rectangles. Three replicate measurements are included in each boxplot (overlayed dots depict the actual values). Phenolics concentrations were measured as mg/ml gallic acid-equivalent.

References

1. McMurdie PJ, Holmes S. phyloseq: An R Package for Reproducible Interactive Analysis and Graphics of Microbiome Census Data. PLOS ONE. 2013;8(4):e61217.

2. Barnett DJ m, Arts IC w, Penders J. microViz: an R package for microbiome data visualization and statistics. J Open Source Softw. 2021;6(63):3201.

3. Dahl EM, Neer E, Bowie KR, Leung ET, Karstens L. microshades: An R Package for Improving Color Accessibility and Organization of Microbiome Data. Microbiol Resour Announc. 2022;11(11):e00795-22.

4. Mandal S, Van Treuren W, White RA, Eggesbø M, Knight R, Peddada SD. Analysis of composition of microbiomes: a novel method for studying microbial composition. Microb Ecol Health Dis. 2015;26(1):27663.

5. Lin H, Peddada SD. Analysis of compositions of microbiomes with bias correction. Nat Commun. 2020;11(1):3514.

6. Andrews S. FastQC: A Quality Control tool for High Throughput Sequence Data [Internet]. Available from: https://www.bioinformatics.babraham.ac.uk/projects/fastqc/

7. Bolger AM, Lohse M, Usadel B. Trimmomatic: a flexible trimmer for Illumina sequence data. Bioinformatics. 2014;30(15):2114–20.

8. Eren AM, Kiefl E, Shaiber A, Veseli I, Miller SE, Schechter MS, et al. Community-led, integrated, reproducible multi-omics with anvi'o. Nat Microbiol. 2021;6(1):3–6.

9. Li D, Liu CM, Luo R, Sadakane K, Lam TW. MEGAHIT: an ultra-fast single-node solution for large and complex metagenomics assembly via succinct de Bruijn graph. Bioinforma Oxf Engl. 2015;31(10):1674–6.

10. Li D, Luo R, Liu CM, Leung CM, Ting HF, Sadakane K, et al. MEGAHIT v1.0: A fast and scalable metagenome assembler driven by advanced methodologies and community practices. Methods San Diego Calif. 2016;102:3–11.

11. Langmead B, Salzberg SL. Fast gapped-read alignment with Bowtie 2. Nat Methods. 2012;9(4):357–9.

12. Martin M. Cutadapt removes adapter sequences from high-throughput sequencing reads. EMBnet.journal. 2011;17(1):10–2.

13. Kang DD, Li F, Kirton E, Thomas A, Egan R, An H, et al. MetaBAT 2: an adaptive binning algorithm for robust and efficient genome reconstruction from metagenome assemblies. PeerJ. 2019;7:e7359.

14. Wu YW, Simmons BA, Singer SW. MaxBin 2.0: an automated binning algorithm to recover genomes from multiple metagenomic datasets. Bioinformatics. 2016;32(4):605–7.

15. Alneberg J, Bjarnason BS, de Bruijn I, Schirmer M, Quick J, Ijaz UZ, et al. Binning metagenomic contigs by coverage and composition. Nat Methods. 2014;11(11):1144–6.

16. Uritskiy GV, DiRuggiero J, Taylor J. MetaWRAP—a flexible pipeline for genome-resolved metagenomic data analysis. Microbiome. 2018;6(1):158.

17. Hyatt D, Chen GL, LoCascio PF, Land ML, Larimer FW, Hauser LJ. Prodigal: prokaryotic gene recognition and translation initiation site identification. BMC Bioinformatics. 2010;11(1):119.

18. Kanehisa M, Sato Y, Morishima K. BlastKOALA and GhostKOALA: KEGG Tools for Functional Characterization of Genome and Metagenome Sequences. J Mol Biol. 2016;428(4):726–31.

19. Shaffer M, Borton MA, McGivern BB, Zayed AA, La Rosa SL, Solden LM, et al. DRAM for distilling microbial metabolism to automate the curation of microbiome function. Nucleic Acids Res. 2020;48(16):8883–900.

20. Brettin T, Davis JJ, Disz T, Edwards RA, Gerdes S, Olsen GJ, et al. RASTtk: A modular and extensible implementation of the RAST algorithm for building custom annotation pipelines and annotating batches of genomes. Sci Rep. 2015;5(1):8365.

21. Arkin AP, Cottingham RW, Henry CS, Harris NL, Stevens RL, Maslov S, et al. KBase: The United States Department of Energy Systems Biology Knowledgebase. Nat Biotechnol. 2018;36(7):566–9.

22. Wilmoth JL, Schaefer JK, Schlesinger DR, Roth SW, Hatcher PG, Shoemaker JK, et al. The role of oxygen in stimulating methane production in wetlands. Glob Change Biol. 2021;27(22):5831–47.
